# Recent Update on PET/CT Radiotracers for Imaging Cerebral Glioma

**DOI:** 10.1007/s13139-024-00847-4

**Published:** 2024-02-21

**Authors:** Dongwoo Kim, Suk-Hyun Lee, Hee Sung Hwang, Sun Jung Kim, Mijin Yun

**Affiliations:** 1 Department of Nuclear Medicine, Severance Hospital, Yonsei University College of Medicine, 50-1 Yonsei-Ro, Seodaemun-Gu, Seoul, 03722 Republic of Korea; 2 Department of Radiology, Hallym University Kangnam Sacred Heart Hospital, Hallym University College of Medicine, Seoul, 07441 Republic of Korea; 3 Department of Nuclear Medicine, Hallym University Sacred Heart Hospital, Hallym University College of Medicine, Anyang, 14068 Republic of Korea; 4 Department of Nuclear Medicine, National Health Insurance Service Ilsan Hospital, Goyang, 10444 Republic of Korea

**Keywords:** Glioma, ^18^F-FDG, Amino acid radiotracer, ^11^C-acetate, ^18^F-FLT, ^18^F-FMISO

## Abstract

Positron emission tomography/computed tomography (PET/CT) has dramatically altered the landscape of noninvasive glioma evaluation, offering complementary insights to those gained through magnetic resonance imaging (MRI). PET/CT scans enable a multifaceted analysis of glioma biology, supporting clinical applications from grading and differential diagnosis to mapping the full extent of tumors and planning subsequent treatments and evaluations. With a broad array of specialized radiotracers, researchers and clinicians can now probe various biological characteristics of gliomas, such as glucose utilization, cellular proliferation, oxygen deficiency, amino acid trafficking, and reactive astrogliosis. This review aims to provide a recent update on the application of versatile PET/CT radiotracers in glioma research and clinical practice.

## Introduction

Cerebral gliomas are the most common form of primary malignant brain tumors, accounting for approximately 80% of such diagnoses [1]. Effective treatment strategies and prediction of patient outcomes are strongly linked to the precise categorization of these tumors, both in terms of histological characteristics and genomic markers. The WHO 2021 update from WHO 2016 incorporated crucial genomic indicators, such as isocitrate dehydrogenase (IDH) mutations, thereby enhancing the classification and prognostic accuracy of these tumors [2, 3]. However, traditional approaches for examining tissue and molecular characteristics typically involve invasive techniques. On the other hand, imaging techniques offer a non-invasive and semi-quantitative alternative, yielding clinically significant data in preoperative settings, which may have important therapeutic implications. [4].

Magnetic resonance imaging (MRI) is currently the gold standard for brain imaging but has its limitations. MRI often faces challenges in clearly distinguishing between low- and high-grade tumors as well as between treatment-induced changes such as radiation necrosis and actual tumor progression [5, 6]. Although it provides limited information about tumor metabolism and molecular features, advanced MRI methods, such as perfusion-weighted imaging, diffusion-weighted imaging, and MR spectroscopy, have been actively investigated in research and clinical settings.

Positron emission tomography/computed tomography (PET/CT) has dramatically changed the landscape of non-invasive evaluation of gliomas. Various PET/CT radiotracers offer insights into a range of biological functions including glucose metabolism, cellular proliferation, amino acid transport, reactive astrogliosis, and hypoxia. PET/CT is invaluable for non-invasive tumor grading, differential diagnosis, prognosis prediction, recurrence evaluation, and monitoring after treatment [5–11]. To further enhance its transformative role and provide guidance for accurately assessing brain gliomas, the field has introduced standardized imaging protocols of various radiotracers [12–14]. This review aimed to provide a comprehensive overview of the PET/CT radiotracers currently used in glioma research and clinical settings. Special focus is placed on radiotracers of clinical and research importance including ^18^F-fluorodeoxyglucose (^18^F-FDG), amino acid-based radiotracers, ^11^C-acetate, ^18^F-fluorothymidine (^18^F-FLT), and ^18^F-fluoromisonidazole (^18^F-FMISO).

## ^18^F-Fluorodeoxyglucose (^18^F-FDG)

The radiotracer had a relatively long half-life of 110 min, allowing it to be transported from the central cyclotron to nearby locations. Radiosynthesis is a relatively straightforward process. Currently, ^18^F-FDG is the most commonly utilized radiotracer for PET/CT imaging in clinical oncology and was first used in the early 1980s for brain tumor imaging [15]. Radiotracers are highly effective in identifying rapidly proliferating cells, as these cells show an increased uptake of ^18^F-FDG. This is largely due to elevated levels of glucose transporters and the enzyme hexokinase, which converts both glucose and ^18^F-FDG into their phosphorylated form [16]. This makes ^18^F-FDG particularly useful for distinguishing high-grade gliomas from other gliomas [7]. Typically, more aggressive tumors demonstrate higher levels of ^18^F-FDG uptake, which has been proven to be a reliable prognostic marker. For example, if a previously identified low-grade tumor starts to show high uptake, it is generally an indicator of the tumor becoming anaplastic [8].

**Fig. 1:**
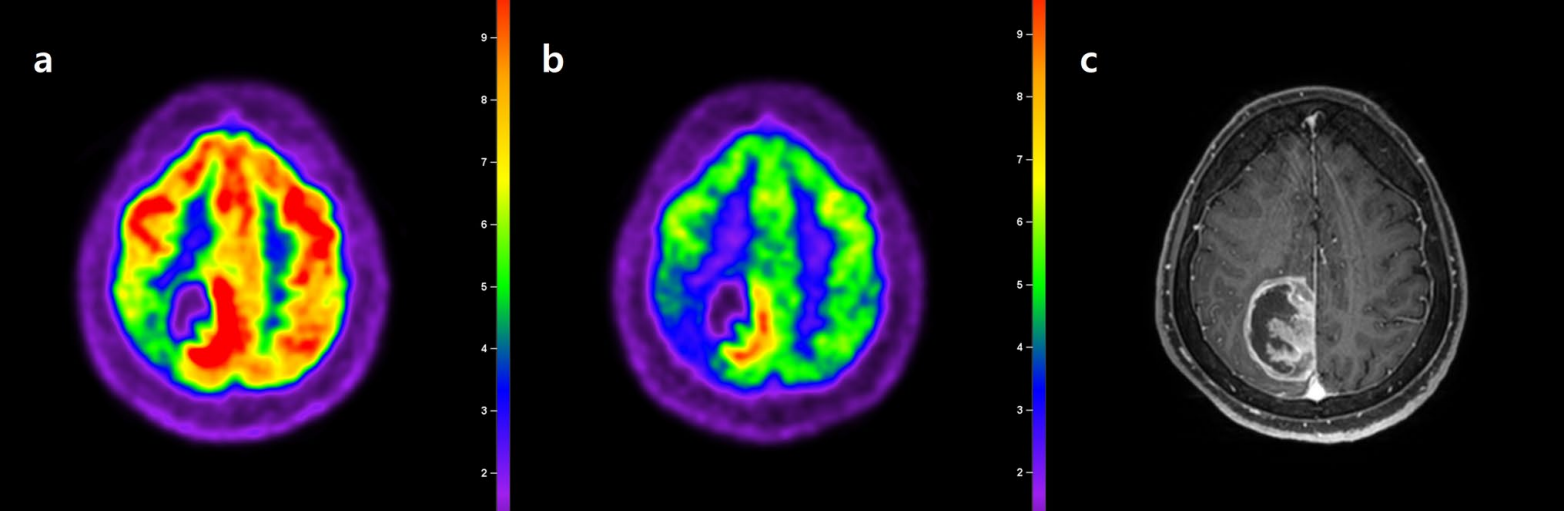
**a** Transaxial ^18^F-FDG PET, **b** glucose-loaded ^18^F-FDG PET, and **c** contrast-enhanced T1-weighted MRI images in a patient with glioblastoma. Due to the high ^18^F-FDG uptake in the normal cerebral cortex, the boundaries of the tumor in the para-sagittal area are not clearly distinguishable in the standard ^18^F-FDG PET scan. However, in the glucose-loaded ^18^F-FDG PET scan, the ^18^F-FDG uptake in the normal cerebral cortex is reduced, allowing for a clearer observation of the tumor’s ^18^F-FDG uptake boundaries

However, recent studies have highlighted the diagnostic limitations of ^18^F-FDG PET/CT. Non-neoplastic neurological diseases, such as bacterial abscesses, tuberculosis, fungal infections, and sarcoidosis, can mimic the appearance of brain tumors on ^18^F-FDG PET/CT scans [17]. Due to the naturally elevated levels of glucose metabolism in normal brain tissue, detecting tumors with only moderate increases in glucose metabolism is challenging [18]. This is particularly problematic for low-grade tumors and, in some cases, for recurrent high-grade tumors. The ^18^F-FDG uptake in low-grade tumors is often similar to that observed in normal white matter, and for high-grade tumors, it might be similar to or even less than the uptake in the normal gray matter. This results in a reduced sensitivity for the detection of tumor lesions. To overcome these limitations, Kim et al. explored the effects of elevated blood glucose levels by administering intravenous glucose before performing ^18^F-FDG PET/CT [19]. Elevated blood glucose levels led to reduced ^18^F-FDG uptake in normal brain tissue, thereby enhancing the ability of the scan to detect gliomas with greater sensitivity (Fig. 1). In a different approach, Johnson et al. focused on the timing of ^18^F-FDG PET scans, demonstrating that a delayed scanning protocol provides superior visibility of glioblastomas compared to conventional timing [20].

A major change in the conventional histology-driven classification of gliomas is the incorporation of genetic alterations. The molecular parameters outlined in the 2016 Central Nervous System (CNS) WHO classification include mutations in the isocitrate dehydrogenase enzyme isoforms 1 (IDH1) and 2 (IDH2), 1p/19q co-deletion, and H3 K27M mutations. Of these, driver mutations in IDH1 and IDH2 genes are involved in the pathogenesis and progression of gliomas, which are genetically classified into IDH mutant and IDH wild-type forms. Cytosolic IDH1 and mitochondrial IDH2 contribute to the production of nicotinamide adenine dinucleotide phosphate (NADPH) from NADP^+^ via oxidative decarboxylation of isocitrate to α-ketoglutarate (α-KG) [21, 22]. IDH mutation-induced NADPH reduction affects cellular defense mechanisms against oxidative stress. In addition, the mutations produce an abnormal metabolite known as 2-hydroxyglutarate instead of α-KG which competitively inhibits α-KG–dependent dioxygenases. The resultant genome-wide epigenetic changes predispose cells to malignant transformations. IDH mutations are found in more than 70% of WHO grade 2 and 3 gliomas and fewer than 10% of glioblastomas. Patients with IDH mutations exhibit longer overall survival (OS) than those with wild-type IDH [23, 24]. ^18^F-FDG PET/CT has been used for in vivo image-guided identification of gliomas with IDH mutations. ^18^F-FDG uptake in IDH1-mutant gliomas is significantly lower than that in IDH1 wild-type gliomas [8]. While IDH1 mutation was the most important factor in identifying patients with the best prognosis, increased ^18^F-FDG uptake provided additional prognostic information for predicting poor OS in patients with IDH1 wild-type gliomas.

## Amino Acid Radiotracers

Amino acids are taken up by the cells through carrier-mediated processes [25]. This forms the basis for amino acid imaging, as multiple studies have documented an increase in amino acid transport during malignant transformation [26, 27]. In experimental animal models, it has been found that increased amino acid transport into tumor cells is facilitated by the upregulation of amino acid transporters in the blood vessels supporting the brain tumor tissue [28]. Amino acid-based radiotracers are particularly promising for brain tumor imaging. They are taken up more readily by tumors and show minimal uptake in the normal brain, thereby offering a high contrast ratio between the tumor and surrounding normal tissue for tumor delineation [29]. Amino acid radiotracers consistently outperform ^18^F-FDG in the diagnosis of brain tumors, particularly low-grade tumors [30–32].

Among these PET/CT tracers, ^11^C-methionine is one of the most important and highly useful for imaging L-type amino acid transporter 1 (LAT-1) [33]. It has been used globally in multiple institutions since the 1980s [33, 34]. Tracers are extensively employed in clinical settings to define the boundaries of brain tumors, staging, prognosis prediction, treatment evaluation, and recurrence identification [4, 33, 35–38]. However, due to ^11^C’s short half-life of 20 min, ^18^F-labeled aromatic amino acid analogs have been developed for tumor imaging. Developed in the late 1990s, 18-fluoride-fluoro-ethyl-tyrosine (^18^F-FET) is an ^18^F-labeled amino acid PET tracer with a longer half-life of 110 min, making it suitable for routine clinical use [39, 40]. Transport inhibition tests with specific competitive inhibitors have shown that more than 80% of ^18^F-FET uptake into cancer cells occurs via an L-type transport system [39]. Unlike other radiotracers, ^18^F-FET is neither incorporated into proteins nor metabolized once it enters the cell and essentially serves as a measure of the amino acid transport rate. Compared to ^18^F-FDG and ^11^C-methionine, ^18^F-FET shows lower uptake in cells related to inflammation, thereby offering greater specificity in distinguishing tumor tissue from inflammation [41–43]. Complementing this specificity, Vidmar et al. have demonstrated the effectiveness of ^18^F-FET PET in distinguishing between treatment-related changes and true progression in glioma patients [44]. In addition to ^11^C-methionine and ^18^F-FET, 3,4-dihydroxy-6-[^18^F]fluoro-l-phenylalanine (^18^F-FDOPA) is another ^18^F-labeled compound initially developed for measuring dopamine synthesis and is primarily used for imaging the basal ganglia [45, 46]. It is mainly transported by LAT1 in tumors and can detect both enhancing and non-enhancing tumors [47]. Additionally, its significance in clinical settings is highlighted by the effective detection of glioma recurrence or progression through the use of ^18^F-FDOPA PET [48].

**Fig. 2:**
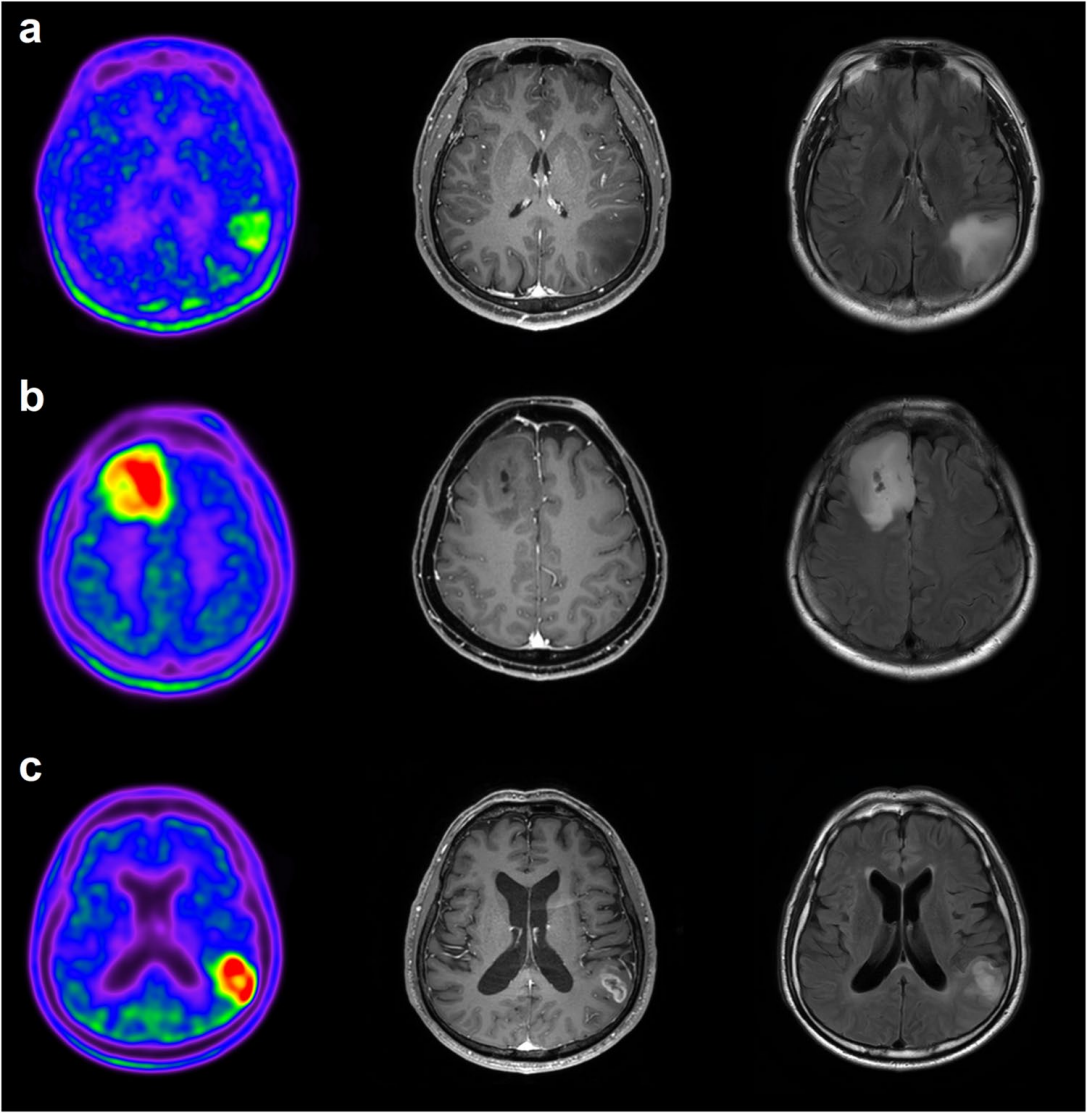
Transaxial ^11^C-methionine PET, contrast-enhanced T1-weighted MRI, and contrast-enhanced T2 FLAIR MRI images. **a** A patient with grade 2 astrocytoma, IDH-mutant. **b** A patient with a grade 3 oligodendroglioma. **c** A patient with glioblastoma. ^11^C-methionine has good sensitivity for detecting low-grade gliomas. However, its uptake in oligodendrogliomas is as high as in glioblastomas, which poses a significant limitation for glioma classification

The 2016 World Health Organization (WHO) classification of cerebral gliomas has led to an improved diagnosis of oligoastrocytomas as either astrocytomas or oligodendrogliomas (OD). Gliomas with IDH1 mutations can be sub-classified into two types: those with 1p/19q co-deletion, known as ODs, and those with intact 1p/19q, identified as astrocytomas, leading to better OS in patients with OD [2]. Based on this improved classification, studies have evaluated amino acid radiotracers for the characterization of amino acid uptake in relation to IDH1 mutations and 1p/19q co-deletion status. Overall, IDH1-mutant gliomas show lower amino acid radiotracer uptake than IDH1-wildtype glioma [49–51]. However, amino acid radiotracer uptake in ODs is as high as that in glioblastomas, which constitutes a limitation of radiolabeled amino acids in glioma classification (Fig. 2). Therefore, amino acid radiotracer uptake for glioma grading may be more consistent in IDH1-wildtype than in IDH1-mutant tumors.

## ^11^C-Acetate

^11^C-Acetate has long been employed as a radiotracer for cardiac oxidative metabolism by measuring clearance rates through the tricarboxylic acid cycle [52]. In the normal brain, acetate functions as an astrocyte-specific substrate and serves as an alternative energy source to glucose [53–56]. The primary mechanism of astrocyte transport and utilization of acetate involves monocarboxylate transporter 1 (MCT1) [54, 57, 58]. Astrocytes are highly sensitive to environmental conditions and undergo dynamic shifts in their molecular, functional, and morphological characteristics in response to various physical and chemical stimuli of the CNS. These astrocytes are known as reactive astrocytes, which can be seen in conditions such as Alzheimer’s disease (AD) and Parkinson’s disease (PD), as well as in patients with stroke and glioblastoma patients [59–63]. Recently, Nam et al. reported increased ^11^C-acetate uptake in reactive astrocytes in animal models of neuroinflammation, as well as in patients with AD [64]. Additionally, there have been two reports on the use of ^11^C-acetate PET/CT for detecting reactive astrocytes in patients with multiple sclerosis [65, 66].

**Fig. 3:**
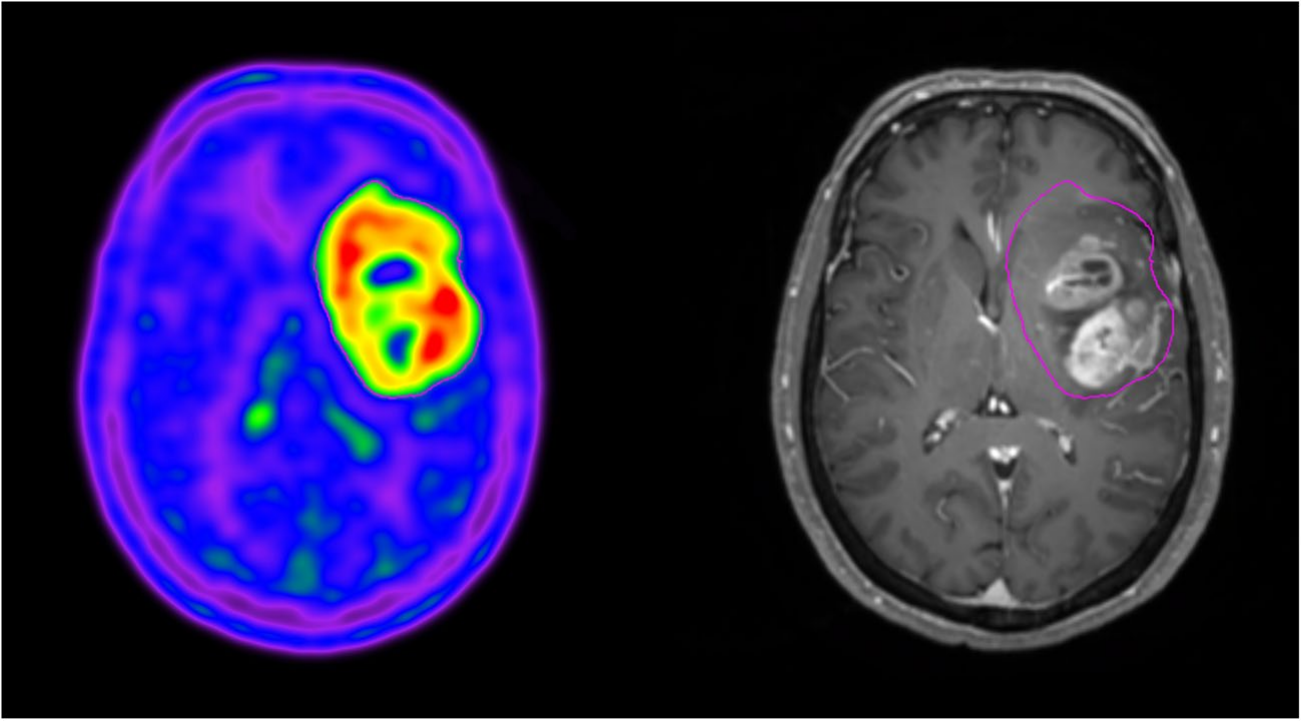
Transaxial ^11^C-acetate PET and contrast-enhanced T1-weighted MRI images in a patient with glioblastoma. Purple demarcation, ^11^C-acetate PET-based tumor margin. Intense ^11^C-acetate uptake is observed in glioblastoma and beyond the tumor boundary

In tumor imaging, ^11^C-acetate serves as a valuable radiotracer for identifying acetate-dependent tumors that cannot be detected using ^18^F-FDG PET/CT. Tumors such as renal cell carcinoma, hepatocellular carcinoma, and well-differentiated prostate cancers exhibit significant ^11^C-acetate uptake, which is ascribed to enhanced lipid synthesis within these tumors [67–69]. In contrast, there are studies implicating acetate as a substrate for lipid metabolism in high-grade gliomas [70, 71]. Using ^11^C-acetate, studies have suggested increased ^11^C-acetate uptake in patients with high-grade tumors [72–76]. ^11^C-Acetate uptake on PET/CT differed significantly between low- and high-grade gliomas and exhibited the capability to further distinguish between grade 3 and grade 4 tumors. ^11^C-Acetate uptake and metabolic tumor volume on PET/CT are independent prognostic factors and predict survival better than the WHO grade [75]. The high ^11^C-acetate uptake associated with higher-grade gliomas is inconsistent with the known finding that ^11^C-acetate is taken up by well-differentiated tumors in the body. This raises the question regarding the cellular origin of ^11^C-acetate in gliomas. In a recent study, conditioned media collected from the IDH1-wt (but not IDH1-mt) human glioblastoma cell line led to the reactivity of mouse primary astrocytes and high ^11^C-acetate uptake [77]. In fact, ^11^C-acetate uptake on PET/CT was discovered to represent reactive astrocytes in the tumor microenvironment (TME) (Fig. 3).

As an important tool to visualize reactive astrogliosis, ^11^C-acetate PET/CT has shown the potential for glioma grading as an important tool for visualizing reactive astrogliosis [78]. As mentioned, radiolabeled amino acids reported unexpectedly high amino acid radiotracer uptake in ODs, similar to the levels observed in high-grade IDH1-wt tumors. Unlike amino acid radiotracers, high ^11^C-acetate uptake was associated with high-grade IDH1-wt tumors, thus facilitating differentiation from high-grade IDH1-mt and low-grade gliomas. In particular, the low ^11^C-acetate uptake in ODs is advantageous for overcoming the limitations of radiolabeled amino acid tracers. In addition, ^11^C-acetate PET/CT appears to have other potential values in evaluating gliomas. First, reactive astrogliosis harbors cancer stem cells and defines the boundaries of advanced tumors in high-grade gliomas. Therefore, ^11^C-acetate PET/CT can be used to determine the surgical margins of tumors. Second, studies have reported conflicting roles of reactive astrogliosis in tumor growth, invasion, and treatment resistance in glioblastoma [79]. ^11^C-Acetate PET/CT can be used as a useful tool to visualize reactive astrogliosis and its effect on tumor growth in vivo following various treatment modifications.

## ^18^F-Fluorothymidine (^18^F-FLT)

^18^F-FLT was initially identified as a selective inhibitor of DNA synthesis [80]. Because thymidine is found only in DNA, the radiolabeled version is expected to indicate the rate of tissue proliferation [81]. ^18^F-FLT is transported into cells through either active nucleoside transporters or simple diffusion [82]. Although it does not integrate into DNA strands, it is trapped in the cell after being phosphorylated by thymidine kinase-1 (TK-1). The activity of TK-1 elevates during the cell cycle’s S-phase and is correlated with tumor growth [81, 83]. Even without becoming a part of the DNA, imaging with ^18^F-FLT demonstrates that cellular uptake occurs, which is related to the levels of Ki-67 expression observed in the resected tumor tissue [84].

For low-grade gliomas, ^18^F-FLT imaging is generally not considered useful because of the minimal uptake of the radiotracer [85]. Tumors with little or no contrast enhancement on MRI also show minimal ^18^F-FLT concentrations, consistent with the established correlation between ^18^F-FLT uptake and contrast enhancement [86, 87]. Generally, high-grade gliomas exhibit high contrast enhancement and ^18^F-FLT uptake, whereas low-grade gliomas do not. The efficacy of ^18^F-FLT in distinguishing high- from low-grade tumors has a sensitivity and accuracy of approximately 92% [88].

## ^18^F-Fluoromisonidazole (^18^F-FMISO)

Hypoxia is characterized by insufficient levels of oxygen, which hamper normal biological processes [89]. Hypoxia is a significant adverse factor affecting patient outcomes, particularly in high-grade gliomas [90]. The primary causes of tumor hypoxia include disrupted blood circulation due to structural and/or functional anomalies, along with rapid tumor expansion, which results in elevated oxygen needs not being met by an adequate supply [91]. Evaluating the degree of hypoxia in tumors is critical, both biologically and clinically, as tumors under hypoxic conditions have shown increased resilience to radiation treatment, heightened chemoresistance, and poor postsurgical prognoses [92].

The first radiotracer developed to detect hypoxia was ^14^C-misonidazole, a beta-emitting agent, introduced in 1981 [93]. This was succeeded by ^18^F-fluoromisonidazole (^18^F-FMISO) [94]. ^18^F-FMISO is a fat-soluble compound belonging to the 2-nitroimidazole class. These compounds enter cells through passive diffusion, and their rates of entry vary based on their fat solubility. When cells are well oxygenated, ^18^F-FMISO can easily exit the extracellular space. However, the reduction process continues under hypoxic conditions, resulting in its accumulation within cells [95]. Given that the level of hypoxia often correlates with the severity and aggressiveness of a tumor, ^18^F-FMISO is considered a valuable tool for identifying high-grade gliomas. Research has shown that ^18^F-FMISO provides a more accurate assessment of the extent of glioblastomas than contrast-enhanced MRI, suggesting its utility in treatment planning [96]. Additionally, hypoxia in the TME triggers the release of factors such as VEGF, which stimulates angiogenesis [97]. Barajas et al. showed that in patients with recurring high-grade gliomas undergoing bevacizumab treatment, there was a noticeable reduction in ^18^F-FMISO uptake [98]. This finding highlights the potential usefulness of ^18^F-FMISO for tracking tumor changes during anti-angiogenic therapies.

## Conclusion

In summary, the evolving landscape of radiotracers in PET/CT is expanding their diagnostic and prognostic capabilities, particularly in oncology. While ^18^F-FDG remains a cornerstone, its limitations in cerebral gliomas have paved the way for specialized radiotracers, such as amino acids, ^11^C-acetate, ^18^F-FMISO, and ^18^F-FLT. Amino acid radiotracers are considered the best because of their high tumor-to-cortical background uptake and their ability to show non-enhancing tumors on MRI. The high amino acid uptake in ODs can be a limitation of radiolabeled amino acids in glioma grading. In contrast, ^11^C-acetate, an astrocyte-specific energy substrate, has significant clinical value in patients with glioma because it allows the visualization of reactive astrocytes in the TME. These advances not only improve diagnostic accuracy but also hold promise for personalized treatment strategies, particularly in patients with glioblastoma. The role of these radiotracers has become even more critical as we move towards more proactive approaches in medicine. Continued research is essential to unlock their full potential in treatment planning and monitoring.

## Data Availability

Data sharing is not applicable to this article, as no datasets were generated or analyzed during the current study.
