# Correction to: Recent Update on PET/CT Radiotracers for Imaging Cerebral Glioma

**DOI:** 10.1007/s13139-024-00879-w

**Published:** 2024-08-27

**Authors:** Dongwoo Kim, Suk‑Hyun Lee, Hee Sung Hwang, Sun Jung Kim, Mijin Yun

**Affiliations:** 1 Department of Nuclear Medicine, Severance Hospital, Yonsei University College of Medicine, 50‑1 Yonsei‑Ro, Seodaemun‑Gu, Seoul, 03722 Republic of Korea; 2 Department of Radiology, Hallym University Kangnam Sacred Heart Hospital, Hallym University College of Medicine, Seoul, 07441 Republic of Korea; 3 Department of Nuclear Medicine, Hallym University Sacred Heart Hospital, Hallym University College of Medicine, Anyang, 14068 Republic of Korea; 4 Department of Nuclear Medicine, National Health Insurance Service Ilsan Hospital, Goyang, 10444 Republic of Korea

**Correction to Nuclear Medicine and Molecular Imaging (2024) 58:237–245**

10.1007/s13139-024-00847-4

A correction to this paper has been published: 10.1007/s13139-024-00847-4.

The article Recent Update on PET/CT Radiotracers for Imaging Cerebral Glioma, written by Dongwoo Kim, Suk‑Hyun Lee, Hee Sung Hwang, Sun Jung Kim and Mijin Yun, was originally published Online First without Open Access. After publication in volume 58, issue 4, pages 237–245 the author decided to opt for Open Choice and to make the article an Open Access publication. Therefore, the copyright of the article has been changed to © The Authors, 2024 and the article is forthwith distributed under the terms of the Creative Commons Attribution (CC BY) license 4.0 International License, which permits use, sharing, adaptation, distribution and reproduction in any medium or format, as long as you give appropriate credit to the original author(s) and the source, provide a link to the Creative Commons licence, and indicate if changes were made. The images or other third party material in this article are included in the article’s Creative Commons licence, unless indicated otherwise in a credit line to the material. If material is not included in the article’s Creative Commons licence and your intended use is not permitted by statutory regulation or exceeds the permitted use, you will need to obtain permission directly from the copyright holder. To view a copy of this licence, visit http://creativecommons.org/licenses/by/4.0.

The original article has been corrected.

